# Pharmacohistory of Cannabis Use—A New Possibility in Future Drug Development for Gastrointestinal Diseases

**DOI:** 10.3390/ijms241914677

**Published:** 2023-09-28

**Authors:** Dinesh Thapa, Leon N. Warne, Marco Falasca

**Affiliations:** 1 Metabolic Signalling Group, Curtin Medical School, Curtin Health Innovation Research Institute, Curtin University, Perth, WA 6102, Australia; dinesh.thapa@postgrad.curtin.edu.au (D.T.); l.warne@lgp.global (L.N.W.); 2 Little Green Pharma, West Perth, WA 6872, Australia

**Keywords:** cannabis, cannabinoids, ethnopharmacology, inflammatory bowel disease, endocannabinoid system, endocannabinoidome, G-protein-coupled receptors

## Abstract

Humans have employed cannabis for multiple uses including medicine, recreation, food, and fibre. The various components such as roots, flowers, seeds, and leaves have been utilized to alleviate pain, inflammation, anxiety, and gastrointestinal disorders like nausea, vomiting, diarrhoea, and inflammatory bowel diseases (IBDs). It has occupied a significant space in ethnomedicines across cultures and religions. Despite multi-dimensional uses, the global prohibition of cannabis by the USA through the introduction of the Marijuana Tax Act in 1937 led to prejudice about the perceived risks of cannabis, overshadowing its medicinal potential. Nevertheless, the discovery of tetrahydrocannabinol (THC), the primary psychoactive compound in cannabis, and the endocannabinoid system renewed scientific interest in understanding the role of cannabis in modulating different conditions, including gastrointestinal disorders. Preparations combining cannabidiol and THC have shown promise in mitigating gut symptoms through anti-inflammatory and motility-enhancing effects. This review revisits the ethnomedicinal use of cannabis in gastrointestinal diseases and emphasizes the need for further research to determine optimal dosages, formulations, and safety profiles of cannabis-based medicines. It also underscores the future potential of cannabinoid-based therapies by leveraging the role of the expanded endocannabinoid system, an endocannabinoidome, in the modulation of gastrointestinal ailments.

## 1. Introduction

Natural products, including herbs, spices, and dietary supplements, have been used in traditional medicine globally, particularly in rural settings, for disease prevention and treatment [1,2,3]. Scientific research has recently focused on validating these traditional uses for new drug discovery to address unmet medical needs and resistance to synthetic drugs [3]. Natural products have been found to possess anti-inflammatory, antioxidant, and immune-boosting properties, potentially preventing diseases such as cancer, heart disease, and diabetes [4,5]. The World Health Organization (WHO) supports the use of natural products in disease prevention and recognizes their role in primary healthcare systems, establishing a global centre for traditional medicine in India. Notably, natural products have contributed to the development of significant drugs like aspirin, artemisinin, and paclitaxel [6,7].

Among various natural products, cannabis has attracted significant interest due to its long history of use in treating conditions like cancer, pain, gastrointestinal disorders, and neurological disorders [8]. In Ayurveda, different herbal preparations of cannabis have been shown to treat a variety of conditions including digestive disorders such as diarrhoea, dysentery, and colitis; respiratory conditions such as asthma, bronchitis, and cough; neurological conditions such as epilepsy, headaches, migraine, and neuropathic pain; psychological conditions such as anxiety, depression, and insomnia; and gynaecological conditions such as menstrual cramps, premenstrual syndrome, and menopause symptoms [9,10,11,12]. Cannabis is also considered sacred in Hinduism and is consumed during the Shivaratri festival for recreational and religious purposes [13]. Additionally, as this festival falls under the winter season, its use has been linked to a therapeutic purpose, i.e., fighting influenza and symptoms of the common cold [14,15,16]. Formulations such as tinctures for cancer, oil massages for pain and inflammation, and seeds for digestive disorders are reported [10].

With advances in science and technologies, research has been conducted to understand the mechanism behind the therapeutic potential of cannabis in different medical conditions. Scientific research has documented the use of cannabis in treating chronic pain, nausea and vomiting, and epilepsy, as well as improving sleep [10,17,18]. Similarly, the use of cannabis for the treatment of digestive disorders has shown to be increasing worldwide [19]. Studies have suggested that cannabinoids may be as effective as existing anti-emetic medicines in reducing chemotherapy-induced nausea and vomiting [20].

This review aims to explore the historical use of cannabis, discuss its ethnomedicinal use in gastrointestinal disorders, elucidate its medicinal and pharmacological role in GI disease, and address current knowledge gaps and challenges in drug development. There is a growing interest in understanding the therapeutic potential of cannabis and cannabinoids and its potential as a treatment option for various medical conditions.

## 2. History of Cannabis Use

China is reported to have been the first country to cultivate cannabis for commercial and medicinal purposes, where the fibres extracted from cannabis bark were used in the manufacturing of clothes, shoes, and paper [21]. Similarly, the first medical use was reported by the Chinese emperor Liu Chi-nu, who reported that crushed cannabis leaves healed his wound. Folklore and anecdotal evidence tell of a farmer observing a snake using cannabis leaves to heal another snake’s wound, leading the farmer to test the plant on his own wound and experience a cure [22]. While these are historical anecdotes, scientific evidence now also supports the effectiveness of cannabis in healing chronic wounds [23,24].

The religious use of cannabis emerged when Hindus recognized it as a sacred plant linked to the god Shiva. Hindu holy books called Vedas mention Shiva bringing a cannabis plant from the Himalayas for enjoyment and meditation [14,16]. It is written in the book that Shiva sought shelter under a tall cannabis plant on a hot day and consumed its leaves, leaving him refreshed, and subsequently adopted cannabis as his favourite herb [22]. Shiva became known as the Lord of Cannabis in Hindu culture, and on the day of Shivaratri, a major Hindu festival, people worship Shiva and consume different forms of cannabis as a sign of devotion. Hindus in Nepal and India also make a recreational drink called “ghotta” using dried cannabis flowers, sugar, and spices mixed in boiling milk. Anecdotal evidence suggests the use of cannabis in treating cold and flu symptoms. Eventually, in the 20th century, the Indian hemp drugs commission concluded that the cannabis plant was an integral part of the Hindu culture and religion, and banning it would bring unhappiness, suffering, and resentment [25]. It is mentioned that cannabis cures fever, dysentery, and sunstroke; clears phlegm; quickens digestion; sharpens appetite; freshens intellect; and gives alertness to the body and gaiety to the mind [25,26].

In Europe, the medical use of cannabis was documented during the Roman Empire, where it was used for industrial purposes and treating medical conditions like earaches, diminished sexual desires, and gout [22]. As people started travelling to different parts of the world, cannabis seeds were carried from China to as far west as the Rhone Valley in France for cultivation.

For this plant with a long history of medicinal, religious, and recreational uses, the USA introduced the Marijuana Tax Act in 1937, leading to widespread misinformation and impacting the use of cannabis for any purpose. The discovery of tetrahydrocannabinol (THC), a primary psychoactive compound in cannabis in 1964, led to the reclassification of the entire cannabis plant as a Schedule 1 drug hindering medical research despite its other uses including food, fibres, and shelters (the basic needs of life).

However, restrictions on cannabis use have been reduced in the past 20 years, with more countries reconsidering its use in various medical conditions based on ethnomedicinal practices and scientific findings. Among the reported ethnomedicinal and traditional uses, the use of cannabis in gastrointestinal disorders has gained prominence globally [8].

## 3. Ethnomedicinal Uses of Cannabis in Gastrointestinal Disorders

The traditional use of cannabis in gastrointestinal illnesses, including diarrhoea, stomach cramps and pain, gastritis, indigestion, nausea, and vomiting, has been frequently reported in various cultures such as Nepal, India, and Pakistan [8]. This has opened potential new therapeutic options for treating chronic GI diseases like inflammatory bowel disease (IBD). Oral ingestion and smoking have been reported as the most common routes of administration for both medicinal and recreational purposes [8]. Dried flowers and leaves are typically smoked, while seeds are taken orally, either raw or roasted.

The documented ethnomedicinal uses of cannabis are summarized in Table 1, highlighting the specific gastrointestinal condition, the parts of the plant used, the formulation, and the countries where its use has been reported.

## 4. Parts of Cannabis Plant Utilised in Traditional Medicines

**Figure 1:**
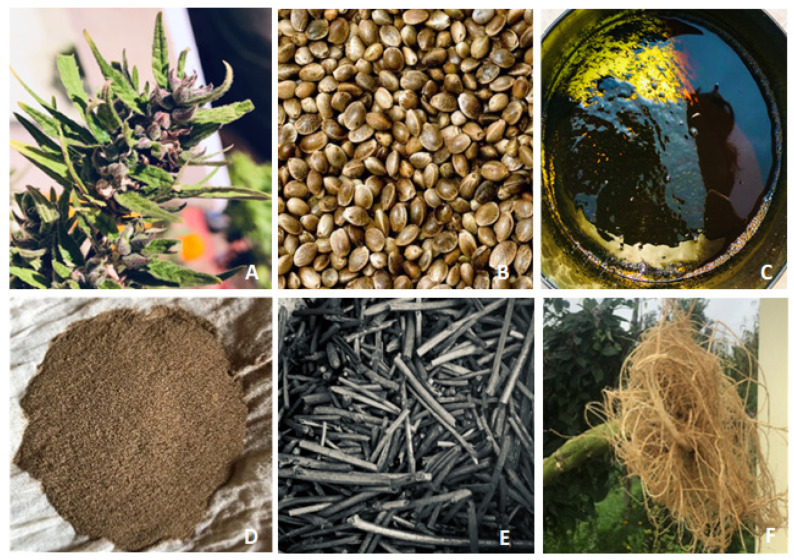
Parts of the cannabis plant reportedly used for medicinal purposes in Nepal. Pictures show different parts of cannabis reported to be used in traditional formulations in Nepal. Flower (**A**), seeds (**B**), extracts (**C**), pollens (**D**), charcoal (**E**), and roots (**F**).

As discussed earlier, the most frequently utilised parts of the cannabis plant reported for medical use are the flowers, seeds, pollens, stem charcoal, roots, and whole extracts (Figure 1). Traditional formulations in the past were based on crude plant preparations or extracts (as shown in Figure 1) as the chemical constituents of cannabis including cannabinoids and terpenes had not been isolated in a pure form.

There are several limitations associated with the use of traditional cannabis formulations in modern clinical practice. One major limitation is a lack of standardization of available formulations for medical use, especially when they are not prepared upon recognized pharmaceutical quality standards. The chemical composition of raw cannabis or extracted traditional formulations can vary significantly due to factors such as cultivars, growing conditions, geographic locations, and processing methods. This variation makes it challenging to ensure a consistent dosing and efficacy across different batches [37].

However, advancements in technology have made it possible to isolate and characterize individual chemical constituents of cannabis. This allows researchers to study the effects of specific components and design new formulations that retain the active cannabinoids and terpenes found in traditional formulations. By optimizing the synergistic ratios of cannabinoids, terpenes, and other phytochemicals, it may enable more precise formulations targeting specific disease management. Additionally, novel delivery technologies such as microencapsulation, nanoemulsions, oral thin films, cannabis-infused edibles, and intranasal sprays are revolutionizing the way cannabis and cannabinoids are consumed, offering improved precision; consistent dosing; a faster onset of action; and improved pharmacokinetics, bioavailability, safety, and user experiences [46,47,48,49,50]. One of the approaches to increase oral bioavailability is through co-administration of cannabinoids with lipids such as dietary fats or pharmaceutical lipid excipients [51]. Oromucosal and nanoparticle-based formulations have also been shown to increase oral bioavailability [52,53]. The carrier vehicle of cannabinoid formulations can also have an impact on oral bioavailability, as demonstrated in a study where the bioavailability of phytocannabinoid THC was enhanced when delivered using sesame oil compared to five different other vehicles [54]. The vehicles used may affect oral bioavailability by modulating first-pass metabolism and degradation in the stomach due to the acidic environment.

## 5. Chemical Profile of Cannabis

**Figure 2:**
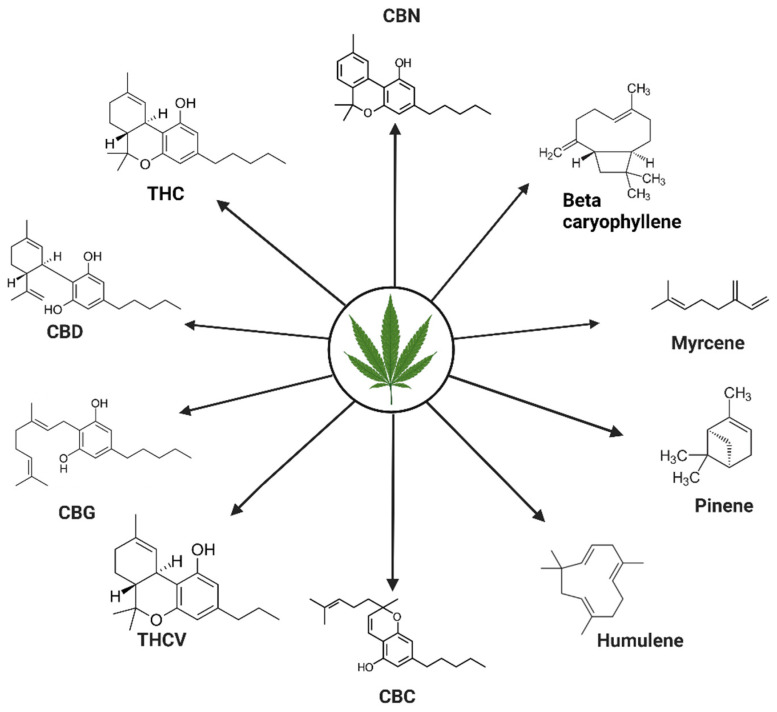
Phytocannabinoids and terpenes present in cannabis plant. The figure shows the chemical structures of some well-studied phytocannabinoids (tetrahydrocannabinol, THC; cannabidiol, CBD; cannabigerol, CBG; tetrahydrocannabivarin, THCV; cannabichromene, CBC; and cannabinol, CBN) and terpenes (beta-caryophyllene, myrcene, pinene, and humulene).

Cannabis contains various chemically active compounds, including cannabinoids, terpenes, alkaloids, and flavonoids. The therapeutic benefits of cannabis primarily come from cannabinoids derived from the plant, known as phytocannabinoids. Terpenes are also purported to contribute to the potential synergistic effects [55,56]. Cannabis is reported to contain approximately 120 phytocannabinoids and 150 terpenes; however, the pharmacology of only a few cannabinoids, such as tetrahydrocannabinol (THC), cannabidiol (CBD), cannabigerol (CBG), cannabinol (CBN), cannabichromene (CBC), tetrahydrocannabivarin (THCV), and terpenes such as pinene, beta- caryophyllene, myrcene, and humulene, have been studied [57,58,59]. Figure 2 shows some of the major phytocannabinoids and terpenes present in the cannabis plant.

Although THC has shown promising results in cellular and animal studies, its therapeutic applications have been limited due to its potential psychotropic effects [60,61,62]. There are, however, several other cannabinoids, such as CBD, which have demonstrated therapeutic benefits without mood-altering side-effects and have gained significant interest in the pharmaceutical and cosmetic industries [63]. Cannabis terpenes, including beta-caryophyllene, myrcene, humulene, and limonene, are also being explored for their therapeutic and commercial potential [64,65,66,67]. When combined with cannabinoids, terpenes have been shown to produce synergistic effects (called the entourage effect) for treating various medical conditions, aligning with the traditional use of the whole cannabis plant, including roots, leaves, flowers, barks, and seeds, rather than isolated cannabinoids. Thus, a combination approach consistent with traditional uses may offer a novel approach to treating specific diseases.

With the discovery of the endocannabinoid system (ECS), specifically the expanded endocannabinoid system (endocannabinoidome), in the human body, it has become easier to explore the complexity and pharmacological role of cannabinoids and terpenes in modulating different diseases and illnesses.

## 6. The Expanded Endocannabinoid System or the Endocannabinoidome

The endocannabinoid system (ECS) has been shown to play a key role in modulating the pharmacological effects of cannabinoids and terpenes. Recently, the endocannabinoidome has been the term used to better define the diverse role of cannabinoids and terpenes within the body and refers to the extended family of ECS.

**Figure 3:**
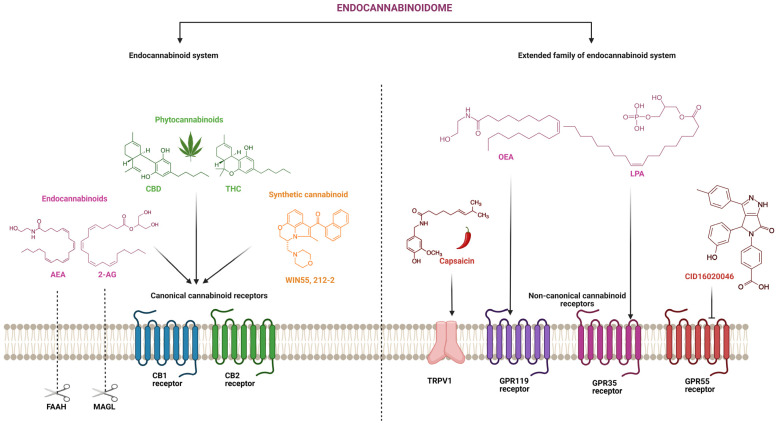
Schematic overview of endocannabinoidome showing classical endocannabinoid system, extended family of endocannabinoid system, and their endogenous and exogenous ligands. Endocannabinoids (anandamide, AEA; and 2-arachidonoylglycerol, 2-AG); phytocannabinoids (cannabidiol, CBD; and tetrahydrocannabinol, THC); synthetic cannabinoid (WIN55, 212-2); endocannabinoid degradation enzymes (fatty acid amide hydrolase, FAAH; and monoacylglycerol lipase, MAGL); canonical cannabinoid receptors (cannabinoid receptor 1, CB1; and cannabinoid receptor 2, CB2); endocannabinoid-like molecules (oleoylethanolamide, OEA; and lysophosphatidic acid, LPA); non-canonical cannabinoid receptors (transient receptor potential of the vanilloid type-1, TRPV1; G-protein-coupled receptor 119, GPR119; G-protein-coupled receptor 35, GPR35; and G-protein-coupled receptor 55, GPR55); TRPV1 agonist (capsaicin); GPR55 antagonist (CID16020046).

The ECS is an important endogenous lipid signalling system that consists of G-protein-coupled receptors (GPCRs), mainly cannabinoid 1 receptor (CB1) and cannabinoid 2 receptor (CB2), and endogenous ligands, i.e., endocannabinoids (eCBs) and the enzymes responsible for the synthesis and degradation of eCBs (Figure 3) [68,69]. Anandamide (AEA) and 2-arachidonoylglycerol (2-AG) are eCBs present in ECS, whereas fatty acid amide hydrolase (FAAH) and monoacylglycerol lipase (MAGL) are their respective catabolic enzymes [69,70]. CB1 receptors are mostly expressed in the central nervous system, i.e., the brain and spinal cord, whereas CB2 receptors are mostly present in immune cells [71,72,73]. The ECS is an emerging therapeutic target for many diseases and disorders including pain, inflammation, gastrointestinal, neurological and cardiometabolic disorders, glaucoma, and cancer [70].

While ECS components including CB1 and CB2, and eCBs and their enzymes have been shown to play a key role in the pharmacological action of cannabis and cannabinoids, recent studies have revealed a more complex picture involving other GPCRs and endocannabinoids-like molecules. GPCRs are the most common human membrane receptor targeted by currently available drugs. More than 34% of FDA-approved drugs target GPCRs and account for annual international sales of over 180 billion USD [74]. GPCRs still have a significant potential as a new therapeutic target as 100 GPCRs, out of 360 endo-GPCRs identified in humans, are still considered orphan receptors [75]. The endocannabinoidome has recently been adopted as a more appropriate term to collectively represent new receptors such as GPCRs—GPR119, GPR55, GPR35, and GPR18; peroxisome-proliferator-activated receptors (PPARs); transient receptor potential of the vanilloid type-1 (TRPV1) channels; endocannabinoid-like molecules such as palmitoylethanolamide (PEA), oleoylethanolamide (OEA), lysophosphatidic acid (LPA), N-acylethanolamines, and N-acyldopamines from the N-acylamides group; and 2-oleoylglycerol and lysophospholipids such as lysophosphatidylinositol (LPI) belonging to the monoacylglycerol group (Figure 3). The expanded family that contains ECS, eCBs, and the extended family of CBRs, a lipid signalling network, is collectively defined as the endocannabinoidome [76].

## 7. The Endocannabinoidome and Inflammatory Bowel Disease

Out of several diseases and disorders affecting the gastrointestinal system, inflammatory bowel disease (IBD) is one of the most challenging and unmet medical needs current healthcare is facing globally. IBDs are chronic conditions that are mainly associated with inflammation of the gastrointestinal (GI) tract. It consists of ulcerative colitis and Crohn’s disease depending on the location of the GI tract involved. Ulcerative colitis is generally an inflammation of the colon whereas Crohn’s disease can affect any part of the GI tract. They are considered a global health problem due to their higher rate of incidence and lack of effective therapeutics [77]. Factors associated with a higher incidence of IBD are often linked to unhealthy diets, smoking, a lack of exercise, pollution, heredity, etc. There is currently no definitive cause associated with IBD; however, genetics and the immune system are thought to be the main driving factors. People of any age can suffer from IBD; however, it is more common among those aged 15 to 40 years [78]. Smokers are twice as likely to suffer from IBD compared to non-smokers. Research has shown that the risk of developing colorectal cancer, stenosis, and fistula is higher in people with active IBD for more than 8–10 years in duration [77]. IBD usually presents with symptoms of GI discomfort such as pain, cramps or swelling of the gut, recurrent diarrhoea or constipation, faecal blood occurrence, and rectal bleeding. However, recent clinical evidence suggests that IBD not only manifest in the GI system but also affects extraintestinal organs such as the joints, eyes, skin, spleen, liver, lungs, and pancreas, leading to several complications such as scleritis, anterior uveitis, ankylosing spondylitis, and peripheral arthritis [79]. Moreover, worsened mental health (anxiety and depression) and the increased incidence of Alzheimer’s disease have also been reported in IBD patients [80,81]. In summary, IBD is a chronic multifactorial disease that requires multiple treatment approaches to maintain an acceptable quality of life.

There is a not a single gold standard treatment for IBD. Current therapies for IBD primarily include aminosalicylates, immunosuppressants, antibiotics, biologics, and surgery [82]. Alternative treatments such as diet plans, lifestyle modifications, and natural medicines are often suggested to ease symptoms and reduce the side-effects associated with pharmacotherapy [83]. Novel therapeutics including anti-tumour necrosis factors, Janus kinase (JAK) inhibitors, and anti-interleukin 12/23 have recently become available to treat the extraintestinal manifestations of IBD; however, the lack of long-term safety data limits the use of these agents in a larger population [84]. Other common challenges observed with current therapies include immunosuppression, metabolic disturbance, hypertension, a loss of response over time, treatment nonadherence, high treatment costs associated with biologics, anti-TNFs, etc. [85,86,87,88]. This clearly highlights the need for a novel therapy that can not only target the GI symptoms but also cure extraintestinal manifestations associated with IBD. The new treatment should also offer lower costs to the patients.

Cannabis and cannabinoids have gained considerable interest because of their medical benefits including antidepressant, analgesic, anti-inflammatory, and immunomodulatory effects [10]. Cannabis has been shown to reduce pain and diarrhoea, increase appetite, and reduce the need for other medications in IBD patients [89,90]. About 15–40% of IBD patients have been shown to use cannabis for reducing their symptoms [91]. Several preclinical studies have also shown that cannabinoids reduce colon inflammation and tissue damage [91,92]. Similarly, endocannabinoids that are produced locally at the intestinal epithelial cells and are transported by p-glycoprotein (p-gp), also called multidrug resistance protein (MDR), into the intestinal lumen are shown to prevent intestinal inflammation and maintain homeostasis. The CB2 receptor has been shown to be involved in these effects as the deletion of the CB2 receptor led to increased neutrophil infiltration in the intestinal lumen, an acute response of inflammation [93]. CB1 receptors are also identified in the colonic epithelium [94]. Rectal administration of endocannabinoids 2-AG using carbon nanotubes as the drug carrier has been shown to improve colitis in the trinitrobenzene sulphonic acid (TNBS) model of colitis, but the effect was absent when 2-AG or carbon nanotubes were administered alone [95]. This suggests that a targeted delivery method could enhance the therapeutic efficacy of the drug. Along with the effect of 2-AG on colitis, intraperitoneal AEA improves TNBS colitis [94]. JZL814, an MAGL inhibitor, has been shown to improve colitis in the TNBS model by reducing mucosal and systemic pro-inflammatory cytokines such as IL-6, TNFα, and IL-12 [96]. Similarly, a study with FAAH inhibition showed similar results, i.e., improved colitis. Genetic knockdown of FAAH produced less severe symptoms [97]. In addition to FAAH and MAGL, the cyclooxygenase-2 (COX-2) enzyme was shown to play a role in interactions between endocannabinoid and prostanoid systems, and the inhibition of COX-2-derived endocannabinoid metabolites such as prostaglandin-glycerol esters (PG-G) and PG-ethanolamides (PG-EA or prostamides) modulates pain and inflammation [98]. The combined blockade of FAAH and cyclooxygenase (COX) activity using PF-3845 and diclofenac respectively has shown increased anti-allodynic effects in neuropathic and inflammatory pain models [99]. FAAH and COX-2 were reported to be elevated in multiple inflammatory conditions including IBD, and the inhibition of these enzymes resulted in the suppression of inflammation and NSAID-induced GI damage [100]. Some recently published studies, including from our own group, have also discussed, in more detail, the involvement and role of the endocannabinoidome in chronic inflammatory intestinal and metabolic diseases such as obesity, diabetes, and IBD [101,102]. In addition to targeting classical cannabinoid receptors, targeting other families of the endocannabinoidome such as FAAH, MAGL, and COX, alone or in combination, could offer novel therapeutics in GI disease and disorders.

Endocannabinoids and phytocannabinoids have demonstrated similar results in a chemical model of colitis as reviewed in a recent study [103]. Oral administration of beta-caryophyllene (BCP), a cannabis terpene, has been shown to improve colitis by acting through CB2 receptors [104]. CBD has been shown to reduce intestinal inflammation (reduction in TNFα) [105] and was shown to potentiate the effect of THC in chemical colitis [106]. The effect of CBD was significant when given intraperitoneally or rectally but not orally [107]. Interestingly, an extract with a high content of CBD given IP or orally reduced tissue damage and intestinal hypermotility in the TNBS-induced IBD model. In the same settings, CBD given alone did not produce a significant improvement in IBD symptoms, suggesting that the CBD shows better action when combined with other minor cannabis constituents [108]. Other phytocannabinoids such as CBG [109], cannabichromene [110], and medicinal cannabis extract [111] have been shown to have beneficial effects on IBD. A recent study has shown that ∆^9^-THC protects against colitis-associated colon cancer via the activation of CB2 receptors [112]. The activation of CB2 receptors is also shown to prevent experimental colitis [113]. Despite the beneficial effects of THC, a recently published study showed that THC exacerbates negative behavioural effects such as anxiety and depression [114].

Taken together, cannabinoids have shown encouraging results in preclinical studies; however, the lack of larger clinical trials, poor medication tolerance, and undesirable side-effects associated with THC are limiting the prescription of cannabinoid medicines in the clinical setting. Some of the reasons associated with the poor translation of preclinical findings could be inappropriate experimental animal models, decreased bioavailability, and poorly targeted drug delivery and pharmacokinetic profiles. Novel formulations and combinational drug delivery strategies targeting different receptors may lead to better outcomes in clinical settings.

## 8. Role of the Expanded Family of ECS in IBD

Along with the classical CB1 and CB2 receptors, other families of GPCRs including GPR119, GPR35, and GPR55 are emerging as novel targets in the treatment of many diseases like cancers and gastrointestinal diseases. GPR35, also considered an orphan receptor, is one of the newest members of GPCRs discovered in 1998. GPR35 has been shown to be expressed in the gastrointestinal tissues mainly in the intestinal crypt enterocytes, colon, and stomach [115]. Two potential endogenous agonists of GPR35 are tryptophan metabolite kynurenic acid (KYNA) and lysophosphatidic acid (LPA). GPR35 demonstrated a protective role in IBD as colitis worsened when GPR35 was genetically knocked out [116]. It was recently shown that the GPR35-expressing macrophages offer a protective role during intestinal inflammation. Moreover, GPR35 maintains intestinal homeostasis by inducing TNF and corticosterone production [117]. While only a few publications describing the role of GPR35 in cancer have been reported, GPR35 could be a novel target in the modulation of gastrointestinal cancers such as colon cancer. Gastric cancer cells have been shown to have a higher expression of GPR35 mRNA than noncancerous gastric mucosa [118], while non-small-cell lung cancer tissue had an overexpression of GPR35 relative to normal lung tissue resulting in drug resistance [119].

Similarly, GPR55 was first isolated and cloned in 1999 and was shown to be abundantly present in brain tissues [120]. It was originally considered an orphan receptor, but recent studies suggested it as a novel putative “type 3” cannabinoid receptor [121,122]. GPR55 has been implicated in the pathophysiology of a variety of diseases and disorders, including gastrointestinal ailments [123,124,125], and inflammatory and neuropathic pain [126]. It has been suggested that GPR55 in the GI tract modulates gut motility and pain associated with inflammation and neuropathy via the mediation of enteric neuron activation and the release of proinflammatory cytokines [125,126]. Increased expression of GPR55 has been implicated in the induction of intestinal inflammation in previous animal studies as well [125]. Suppressing GPR55 either by using an antagonist CID16020046 (Figure 3) or through genetic knockdown of GPR55 significantly reduced intestinal inflammation by suppressing inflammatory cytokines, and inhibiting leukocyte activation and migration [127]. These preclinical findings were also supported by a clinical trial conducted by Włodarczyk et al., 2017, which reported that patients with IBD had a higher mRNA expression of GPR55 compared to healthy patients. It was also shown that the concentration of GPR55 was elevated in the inflamed colon compared to non-inflamed colonic tissues, suggesting their proinflammatory roles [128]. It has been reported that inflammation and neuropathic hypersensitivity were absent following the genetic knockdown of GPR55 in a mouse model of mechanical hyperalgesia using Freund’s complete adjuvant [129]. GPR55 was also shown to have crosstalk with CB2 receptors in neutrophil chemotaxis and recruitment [130]. It has been reported that both CB1 and CB2 receptor activation has an anti-inflammatory role and inhibits neutrophils’ recruitment [131,132]. Cannabinoid therapeutics that can target both CB2 and GPR55 receptors could offer additive/synergistic effects in IBD.

Taken together, GPR35 and GPR55 influence the IBD and cancer features such as regulation of inflammation, pain, metabolic changes, and hypoxia [133,134]. GPR55- or GPR35-specific ligands could offer novel potential therapeutics for IBD patients.

## 9. Clinically Available Cannabinoids-Based Drugs

The increased research interest in cannabinoids and the endocannabinoid system has led pharmaceutical companies to develop some cannabinoid-based drugs for human use, with one of the most recent examples being Epidiolex^®^ in 2018. Epidiolex is an oral drug based on CBD used for the treatment of seizures associated with two rare and severe forms of childhood epilepsy. Additionally, the FDA has approved synthetic cannabinoid-based drugs called Dronabinol and Nabilone to treat nausea and vomiting associated with cancer chemotherapy in individuals who have developed resistance to other medications. Dronabinol is also approved to treat loss of appetite and weight loss in people with AIDS. Dronabinol contains delta-9-THC, while Nabilone contains a semi-synthetic substance with a chemical structure similar to THC. In 2016, the FDA also approved Syndros, a liquid form of Dronabinol. Another drug called Nabiximols, also known as Sativex, developed by the UK company GW Pharmaceutical, was approved in the United Kingdom in 2010 as an oral mouth spray for treating neuropathic pain, spasticity, overactive bladder, and other symptoms of multiple sclerosis. The active ingredients of this drug are THC and CBD, which are standardized in an approximate ratio of 1:1 so that a single spray delivers a dose of 2.7 mg of THC and 2.5 mg of CBD.

While only a few drugs are currently available on the market, many more are in various stages of pre-clinical and clinical development.

## 10. Clinical Trials Investigating the Use of Cannabis and Cannabinoids in GI Disorders

The clinical trials, investigating cannabis and cannabinoids in the treatment of IBD (UC and Crohn’s), have shown mixed results. While some studies have shown promising results in terms of symptom management and quality of life, others have not found significant differences between cannabinoid treatments and placebos in achieving clinical remission. A list of the clinical trials reported in clinicaltrials.gov is shown in Table 2, which summarizes the key findings.

The majority of clinical trials conducted so far have used either whole cannabis extracts or THC and CBD, alone or in combination. Uses of other phytocannabinoids, cannabis parts, and formulations have not been reported.

## 11. Conclusions and Future Perspectives

The safe use of traditional cannabis formulations for treating various illnesses, especially GI disorders, has been reported worldwide over a long period time. No toxic effects have been reported, except for some hallucinogenic and behavioural effects, which are mostly dependent on the chemical constituents, dosage, and routes of administration. With the recent understanding of the endocannabinoid system as a novel therapeutic target in modulating several diseases, researchers have started delving into the complex mechanisms underlying the therapeutic effects of cannabis and cannabinoids in greater detail. This has led to the development of cannabinoid-based drugs such as Sativex and Epidiolex by GW Pharmaceutical for clinical use in the human population. This has also attracted other pharmaceutical companies like Pfizer (USA) and Tetra Bio-Pharma (Canada) to invest in research on cannabinoid-based drug discovery. Pfizer’s focus is on modulating the CB2 receptor as a novel target for treating inflammatory conditions, while Tetra Bio-Pharma is working on various conditions including sepsis, COVID-19, cancer pain, ocular disorders, and hepatocellular carcinomas.

**Figure 4:**
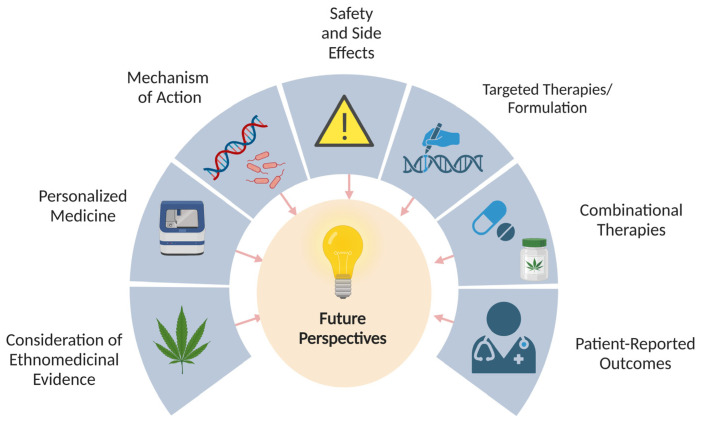
Future research perspectives on cannabis and cannabinoids in GI disorders.

Despite encouraging findings in preclinical and in vitro research, only a few clinical trials have been conducted so far. Traditional formulations such as the oral administration of dried cannabis powders in managing GI symptoms such as diarrhoea and abdominal cramps are not being considered in present human trials. Similarly, most of the trials are solely focused on THC- or CBD-dominant cannabis extracts, and the therapeutic application of THC is limited due to their CNS-related side-effects. Therefore, future cannabis/cannabinoid-based drug discovery research should focus on targeted drug delivery techniques to avoid off-target CNS-related side-effects. Rimonabant, a synthetic anti-obesity drug developed by Sanofi pharmaceutical company, which works by blocking the CB1 receptors, was withdrawn from the market due to CNS-related mood-altering psychoactive side-effects despite demonstrating a positive impact on weight reduction [142]. The use of peripherally restricted blockers targeting CB1 receptors in the gut could minimise such central effects. Similarly, another clinical trial, also called the BIA 10-2474 trial, investigating the safety and efficacy of a synthetic FAAH inhibitor BIA-10-2474, resulted in a tragic outcome with a fatality and severe irreversible neurological symptoms. While the precise cause of this incident is still a matter of investigation and debate, several conclusions were made such as the safety of the drug BIA 10-2474, complexity of ECS, drug-induced off-target effects, trial design, and regulatory oversights [143,144]. With respect to the use of traditional cannabis and phytocannabinoids-based formulations in managing GI symptoms, further research should be focused on investigating the role of the endocannabinoidome in mediating such beneficial effects. The effects of different cannabis cultivars in reducing inflammation and pain, and modulating gut motility and the immune response could be further explored by studying the role of individual compounds in the modulation of the endocannabinoidome. Figure 4 highlights future research perspectives on cannabis and cannabinoids research in GI disorders.

The following points highlight potential future directions for cannabis and cannabinoids research in the treatment of GI disorders.

### 11.1. Consideration of Ethnomedicinal Evidence

Future work should also consider the long history and ethnomedicinal uses of cannabis and cannabinoids throughout the globe. The utilization of traditional formulations while designing new research and clinical trials could result in better outcomes.

### 11.2. Personalized Medicine

Cannabis and cannabinoids have been found to be safe and effective in some population while possessing risk in others. Advancements in genetics and precision medicine may allow for the identification of biomarkers that predict individual response to cannabinoid treatments. Changes in gut microbiomes have been linked with several diseases including inflammatory bowel disease. Understanding the presence or absence of certain microbiome profiles in the GI could help in choosing the best personalized treatment. This personalized approach can help tailor therapies to specific patient profiles, optimizing treatment outcomes for gastrointestinal disorders.

### 11.3. Further Understanding the Mechanisms of Action

Most of the previously published research has focused on CB1 or CB2 receptors as a potential target for the modulation of IBD, leading to unclear mechanisms of action. Further investigation is needed to understand the underlying mechanisms through which cannabis and cannabinoids exert their therapeutic effects on the gastrointestinal system. This includes exploring their interactions with the receptors of the endocannabinoidome family such as GPR55, GPR35, GPR119, GPR118, inflammatory pathways, gut microbiota, and other relevant biological targets.

### 11.4. Safety and Side-Effects

The optimal dosing and delivery methods including oral, topical, and inhalation should be investigated. The dosing and delivery methods of cannabinoids can greatly affect their efficacy and safety. GI-specific novel formulations and delivery technologies can minimize the off-target effects. Future research could explore the optimal dosing and colon-specific delivery methods for different cannabinoids.

### 11.5. Targeted Therapies Exploring the Effects of Different Cannabinoids

Most clinical trials have focused on the effects of THC and CBD. Future clinical trials could investigate the effects of other cannabinoids, such as tetrahydrocannabivarin (THCV), cannabigerol (CBG), and cannabichromene (CBC), alone or in combination in different GI disorders such as IBD, IBS, and GI motility disorders.

### 11.6. Studying the Effects of Cannabinoids in Combination with Other Treatments

Given the complex nature of GI diseases and IBD, research should explore the potential benefits of using cannabinoids in combination with other existing treatments, such as anti-inflammatory drugs, probiotics, or dietary interventions. Clinical trials involving CBD alone were not effective in managing colitis and remission. Some studies have suggested that cannabinoids may have additive or synergistic effects when used in combination with other treatments, such as immunomodulators or biological agents. Future research could investigate the potential benefits of combining sub-therapeutic doses of existing IBD treatments with cannabinoids such as CBD.

### 11.7. Patient-Reported Outcomes

Future research should also focus on collecting evidence from patient-reported outcomes, such as quality of life and symptom relief, to understand the real-world impact of cannabis and cannabinoids on individuals with GI diseases and IBD. Previous studies with cannabinoids have focused on the effects on symptom management and clinical remission. Future research could investigate the potential effects of cannabis and cannabinoids on disease progression by reporting long-term outcomes in IBD. As research progresses, there is also a need to educate healthcare professionals, patients, and the public about the potential benefits, risks, and regulatory landscape surrounding cannabinoid-based therapies for gastrointestinal disorders. Raising awareness can help facilitate informed decision making and reduce stigma.

Taken together, the future of cannabis and cannabinoids research for gastrointestinal disorders involves a comprehensive understanding of their mechanisms of action, multi-centred rigorous clinical trials, personalized medicine approaches, and continued exploration of formulation development and safety considerations. These efforts have the potential to yield novel therapeutic options and improve the quality of life for patients with gastrointestinal disorders.

## Figures and Tables

**Table 1:** Ethnomedicinal uses of cannabis for treating GI disorders.

GI Condition	Parts Used	Preparation/Formulation	Country	Reference
Abdominal cramps and pain	In, Se	A pinch of seed and/or flower powder taken orally with hot water	Nepal	[27]
Abdominal cramps and pain	L, Se,	Crushed dried flowers and seeds taken orally alone or with foods/water	Nepal	[28]
Abdominal pain Antidiarrheal/Dysentery	Se, whole	Paste made from raw seeds/oral	Nepal	[28,29]
Antidiarrheal	Se	Fried seed in cow ghee taken orally	Nepal	[30]
Antidiarrheal, dysentery	In, Se	Dried leaves/flowers with milk	Nepal	[31]
Antidiarrheal	L, whole plant	Leaves juice/seeds	Nepal	[32]
Gastritis	Se	Seed oil/Seed chutney	Nepal	[33]
Appetite stimulant	In, L, Se	Mixed juice/orally	India, Nepal	[34,35]
Severe Stomach pain and wound healing	Resin	Small quantity of Resin/charas/orally	India	[36,37,38]
Indigestion	Rt	About 1 cm root chewed after dinner for 5 days	India	[39]
Constipation	Se	Administered orally in the form of decoction	China	[40,41]
Stomach and liver inflammation	L	Fresh leaf extract with sugar taken orally once in the morning	Pakistan	[42]
Stomach Pain	In, L, St	Dried powder is smoked	Morocco	[43]
Weight Loss	L	Fresh leaves are crushed, soaked in water, and extract is mixed with vinegar	South Africa	[44]
Stomach Pain	Rt	Boiled roots taken orally	Philippines	[45]

Abbreviations: In—inflorescence; L—leaf; Se—seed; Rt—root; St—stem.

**Table 2:** Clinical trials on the use of cannabis and cannabinoids in IBD reported in https://clinicaltrials.gov/ (accessed on 20 July 2023) showing ongoing/completed studies. Irritable bowel syndrome, IBS; inflammatory bowel disease, IBD; cannabidiol, CBD; tetrahydrocannabinol, THC; Crohn’s disease, CD; ulcerative colitis, UC.

Study/NCT Trial Number	Participants/Enrolment	Intervention	Results
NCT01253408	75 (IBS)	2.5 mg or 5 mg dronabinol and placebo	Reduced colonic motility [135]
[136]	13 (IBD)	Cannabis oil containing CBD and THC	Improvement in clinical activity index, body mass index (BMI), and quality of life
NCT01040910	21 (Crohn’s)	Cannabis cigarettes containing 115 mg of THC	Improvement in clinical activity index and improved quality of life [137]
NCT01037322	20 (Crohn’s)	Low-dose CBD (10 mg) or placebo orally twice a day	CBD found to be safe, but no beneficial effects compared to placebo [138]
NCT01562314	60 (UC)	One to five 50 mg CBD capsules taken twice a day	CBD-rich botanical extract found to be beneficial for symptomatic treatment of UC [139]
[140]	56 (Crohn’s)	CBD-enriched cannabis oil or placebo	Significant improvement in clinical parameters and quality of life but no change in inflammatory and endoscopic parameters between groups [140]
NCT01040910	32 (UC)	Cannabis cigarettes (0.5 g of dried flowers with 80 mg of THC) or placebo	Clinical remission and improved quality of life but no association with anti-inflammatory markers [141]
NCT03467620	36 (Crohn’s)	25 mg of CBD capsules/day for 12 weeks	Study withdrawn due to insufficient funds
NCT04056442	28 (Crohn’s)	Synthetic CBD up to 300 mg/day to measure the safety, tolerability, and efficacy in steroid-dependent Crohn’s disease	Ongoing/patients recruiting
NCT05578313	1000 (UC, Crohn’s, pouchitis)	Clinically prescribed medical cannabis or healthy patients	Ongoing/patients recruiting
NCT03886532	Aimed to treat IBD, neuropathic pain	IBD patients taking medical cannabis or CBD legally	Study withdrawn due to insufficient funds
NCT03422861	80 (IBD)	Nabilone 1 mg BID orally or placebo	Not yet recruiting
NCT04055662	100 (effective IBD bowel surgery)	Study the post-operative analgesia requirement in recreational cannabis users vs. non-cannabis users and IBD patients	Results not published yet
NCT01826188	50 (CD)	THC 5mg/mL and CBD 50mg/mL in olive oil taken BID	Patients recruited

## Data Availability

Not applicable.
